# Effects of air pollution caused by sugarcane burning in Western São Paulo on the cardiovascular system

**DOI:** 10.1590/S1518-8787.2017051006495

**Published:** 2017-02-23

**Authors:** Paula Roberta da Silva Pestana, Alfésio Luís Ferreira Braga, Ercy Mara Cipulo Ramos, Ariadna Ferraz de Oliveira, Christian Robert Osadnik, Aline Duarte Ferreira, Dionei Ramos

**Affiliations:** I Departamento de Fisioterapia. Faculdade de Ciências e Tecnologia. Universidade Estadual Paulista Júlio de Mesquita Filho. Presidente Prudente, SP, Brasil; II Departamento de Patologia. Núcleo de Estudos em Epidemiologia Ambiental. Laboratório de Poluição Atmosférica Experimental. Faculdade de Medicina. Universidade de São Paulo. São Paulo, SP, Brasil; III Programa de Pós-Graduação em Saúde Coletiva. Universidade Católica de Santos. Santos, SP, Brasil; IV Departamento de Fisioterapia e Educação Física. Universidade do Oeste Paulista. Presidente Prudente, SP, Brasil; V Department of Rehabilitation Sciences. Katholieke Universiteit Leuven. Leuven, Belgium; VI Department of Physiotherapy. Monash University. Frankston, Victoria, Australia; VII Institute for Breathing and Sleep. Heidelberg, Australia

**Keywords:** Nitrogen Dioxide, adverse effects, Air Pollution, adverse effects, Agricultural Cultivation, Fires, Cardiovascular Diseases

## Abstract

**OBJECTIVE:**

To evaluate the effects of acute exposure to air pollutants (NO_2_ and PM_10_) on hospitalization of adults and older people with cardiovascular diseases in Western São Paulo.

**METHODS:**

Daily cardiovascular-related hospitalization data (CID10 – I00 to I99) were acquired by the Department of Informatics of the Brazilian Unified Health System (DATASUS) from January 2009 to December 2012. Daily levels of NO_2_ and PM_10_ and weather data were obtained from *Companhia Ambiental do Estado de São Paulo* (CETESB – São Paulo State Environmental Agency). To estimate the effects of air pollutants exposure on hospital admissions, generalized linear Poisson regression models were used.

**RESULTS:**

During the study period, 6,363 hospitalizations were analysed. On the day of NO_2_ exposure, an increase of 1.12% (95%CI 0.05–2.20) was observed in the interquartile range along with an increase in hospital admissions. For PM_10_, a pattern of similar effect was observed; however, results were not statistically significant.

**CONCLUSIONS:**

Even though with values within established limits, NO_2_ is an important short-term risk factor for cardiovascular morbidity.

## INTRODUCTION

Human exposure to air pollutants from anthropogenic sources is a public health problem and contributes to increased morbidity due to cardiovascular disease^3,9^. Primary pollutants, such as particulate matter (PM_10_ and PM_2,5_), sulfur dioxide (SO_2_), nitrogen dioxide (NO_2_) and carbon monoxide (CO), have been shown to have significant negative effects on the cardiovascular system. They are also known triggers for a range of cardiovascular-related conditions such as angina pectoris, acute myocardial infarction, thromboembolic complications, arrhythmias, and decompensated congestive cardiac failure^18^. Therefore, it is reasonable to hypothesize that sustained exposure to air pollutants may relate to the incidence of hospitalization for cardiovascular-related conditions.

This rationale is supported by evidence of association between raised air NO_2_ levels and the development of cardiovascular disease^21^ and significant increases in the risk of hospitalization due to myocardial infarction (mean increase of 12.7% [95%CI 5.8–18)^14^. Data also exist from large (n > 400,000 people) international datasets^23^; however, extrapolation of findings beyond the region of the original investigations can be difficult. For example, differences in air pollution matter can occur between metropolitan and rural regions, while differences in source space-time variability can occur between similar metropolitan regions of different geographical regions. Importantly, regional exposure to air pollutants, even in the short-term or in response to low concentrations, is strongly associated with poor health outcomes^22^. Climatic factors, such as thermal temperature, may amplify this response, since hotter weather has been considered a causative factor for increased cardiovascular-related hospitalization episodes in developed^12,20^ and developing countries^2^.

São Paulo city is a well-known, densely populated metropolis of Brazil and a major center for a wide range of industries. Its heavy reliance upon significant vehicular fleet predisposes it to be a region of dense air pollution, posing potential health risks to the general community and those who may be susceptible to the effects of air pollutant exposure such as those with cardiovascular disease^19^. The Western region of São Paulo state, however, has unique occupational and environmental concerns, since it is the center of a major sugar mill industry, estimated to produce and harvest approximately 60.0% of the nation’s total sugarcane^8^. A major public health concern associated with this industry is the necessity for plantation burning, conducted to increase productivity and safety of sugarcane cutters. This phase is associated with a rapid surge in release of particulate matter and toxic gases into the environment of regions where sugarcane is planted, harvested, and industrialized.

Potential health issues related to this exposure may be far-reaching from these plantation sites. For example, traces of matter have been shown to travel (via direct and indirect means) to places as far as Presidente Prudente, SP^2,5^. Such far-reaching effects of excessive PM_10_ levels and their associated negative impact on cardiovascular health have been observed in other parts of South America, with a recent study from Mexico showing detection of air pollution as far as a 500 km radius of the original source^4^.

It is of high importance that the relationship between local exposure to air pollutants and cardiovascular-related health outcomes is further elucidated, particularly in developing countries, such as Brazil, and regions such as Western São Paulo, where the likelihood of adverse health effects may be increased. The purpose of this study was to determine the relationship between acute exposure to air pollution (NO_2_ and PM_10_) and hospitalization of adults and older adults with cardiovascular diseases residing in Western São Paulo, Brazil.

## METHODS

This is an ecological time-series study in the city of Presidente Prudente, Western São Paulo (latitude 22º07’32”S longitude 51º23’20”W). Presidente Prudente is situated 587 kilometers from the capital city, and occupies a total area of 562 km^2^. Its population of 220,600 inhabitants makes it the 34th biggest city in São Paulo state, according to IBGE^a^.

Daily data of adults and older adults’ (19 years of age or older) hospitalizations due to cardiovascular diseases (International Classification of Diseases – ICD, 10th revision I00 to I99) in hospitals of the Brazilian Unified Health System (SUS) were obtained from January 2009 to December 2012. Data were included in the study if hospitalizations occurred with residents of Presidente Prudente. Daily concentrations of air pollutants particulate matter (PM_10_) and nitrogen dioxide (NO_2_), and daily values of temperature, relative humidity, precipitation, and wind speed were obtained from *Companhia Ambiental do Estado de São Paulo* (CETESB – São Paulo State Environmental Agency). Since data can only be collected from one monitoring station, located in the city (in the *campus* of Faculdade de Ciências e Tecnologia – UNESP), measurements of air pollutants and weather were considered representative of the whole city for the purpose of this study.

Descriptive analyses were carried out for all variables in the study. Correlations between pollutants and meteorological variables were estimated using Pearson’s or Spearman’s correlation coefficients. The effect of pollutant exposure on cardiovascular hospitalizations was determined via generalized linear Poisson regression models, in which the daily number of cardiovascular hospitalizations was considered the dependent variable, and daily mean levels of each pollutant concentration (PM_10_ and NO_2_), the independent variables. Seasonality was controlled using natural cubic splines to account for the nonlinear dependence of the dependent variable on time (days) and to exclude the basic seasonal pattern from data. We used 16 degrees of freedom to smooth the time trend minimizing both the autocorrelation between the residuals and the Akaike Information Criterion^11^. After adjusting for the time trend, no remaining serial correlation was found in the residuals, and therefore the use of autoregressive terms was unnecessary. Indicators for day of the week were included to control short-term trends. Since temperature in Presidente Prudente is usually high throughout the year, linear terms for temperature and relative humidity were adopted. To reduce sensitivity to outliers in the dependent variable, robust regression (M-estimation) was used. The lag structure of PM_10_ and NO_2_ effects on cardiovascular hospital admissions from the same day to six days before admission was tested using a third degree polynomial distributed lag model^5^. This approach imposes constraints, but provides enough flexibility to estimate a biologically plausible lag structure controlling for multicollinearity that is better than an unconstrained lag model. Standard errors of the estimates for each day were adjusted for overdispersion.

Effects of air pollutants were expressed as a percent increase and 95% confidence intervals in cardiovascular-related hospitalizations interquartile range (the variation between the 75.0% higher and the 25.0% lower daily concentrations) increases in pollutants concentrations. All data were prepared using Statistical Package for Social Science (SPSS) program for Windows, version 17.0. Analyses were performed via S-PLUS 2000 program for Windows. The Project was approved by the Research Ethics Committee of Faculdade de Ciências e Tecnologia (UNESP), Presidente Prudente *campus*, São Paulo, Brazil (Protocol 51922).

## RESULTS

Table summarises the descriptive analyses of all variables included in the study. Throughout the study period, a total of 6,363 hospitalizations were recorded and attributed as being caused by a cardiovascular condition. Almost half of these hospitalizations, comprising 2,846 cases (44.7%), were related to people aged 20 to 60, while 3,561 (55.3%) were from individuals aged above 60 years. The mean number of hospitalizations per day attributable to cardiovascular diseases increased over time during the study period. From 2009 to 2012, these numbers were 0.55 (SD = 0.4), 0.58 (SD = 0.5), 0.60 (SD = 0.5) and 0.59 (SD = 0.5), respectively.

**Table:** Descriptive analysis of mean daily cardiovascular-related hospitalizations. Western São Paulo state, Brazil, 2009-2012.

Variable	Mean	SD	Minimum	Maximum	Percentiles		

					25th	50th	75th
Total CVD hospital admissions	4.4	2.5	0	16.0	3	4	6
CVD hospital admissions for people aged 20-60 years	1.10	1.7	0	12.0	1	2	3
CVD hospital admissions for people aged > 60 years	2.4	1.8	0	12.0	1	2	3
Pollutants							
PM_10_	18.9	13.9	0	101.3	9.41	15.6	26.2
NO_2_	40.7	27.9	0	146.0	21.0	37.0	58.0
Meteorological variable							
Minimum temperature (°C)	18.8	4.5	0	27.8	17.2	19.3	22.0
Mean temperature (°C)	23.3	3.4	8.80	32.8	21.5	23.9	25.6
Relative humidity (%)	64.9	13.8	15.00	98.5	55.5	65.5	75.0
Wind speed (m/s)	1.6	0.6	0.20	3.8	1.2	1.6	2.1
Wind direction (º)	132.4	49.3	10.16	318.1	97.8	119.3	159.0
Precipitation (mbar)	1.3	6.2	0	110.8	0	0	0.2

CVD: cardiovascular disease; PM: particulate matter; NO_2_: nitrogen dioxide

**Figure 1:**
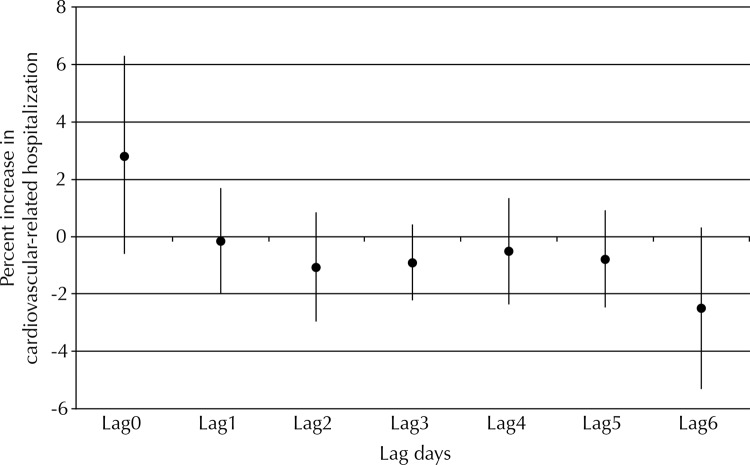
Relationship between percent increase (95%CI) in cardiovascular-related hospitalizations and PM10 exposure. Western São Paulo state, Brazil, 2009-2012.

On average, the recorded levels of air pollutants PM_10_ and NO_2_ remained within the acceptable limits of air quality according to the World Health Organization (WHO) on most observation days. Daily levels of PM_10_ second WHO recommended levels (50 µg/m^3^) on any days remaining on average at 64.25 µg/m^3^. Daily levels of PM_10_ were not significantly related to cardiovascular-related hospitalizations (Figure 1).

Lag: days of exposure; lag0: same day; lag1-6: one to six days after exposure.

The average temperature and relative humidity were related to hospitalizations and air pollutants (NO_2_ and PM_10_), but were not significant during the study period.

**Figure 2:**
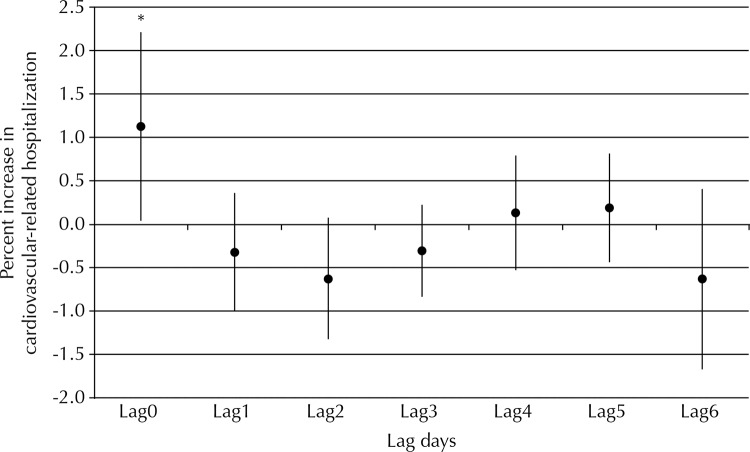
Lag days between percent increase (95%CI) in cardiovascular-related hospitalizations and NO2 exposure. Western São Paulo state, Brazil, 2009-2012.

For NO_2_, the WHO recommended acceptable daily limit (100 µg/m^3^) was exceeded after 37 days, with an average level of 113.11 µg/m^3^ during the study period. Levels of NO_2_ were statistically significantly associated with increased risk of hospitalization due to cardiovascular disease, but only on the day that excessively raised NO_2_ levels were detected (lag 0d) (percent increase of 1.12% [95%CI 0.05–2.20]). This significant, short-term effect was of a small magnitude, and tended to reduce over the subsequent six days (to lag 6d). This positive relationship and trend over time was observed in both the younger adult group (20-60 years) and the older patient group (> 60 years) (Figure 2).

Lag: days of exposure; lag0: same day; lag1-6: one to six days after exposure.

* p < 0.05

## DISCUSSION

This study is the first of its kind to show that indirect exposure to excessive NO_2_ air pollution levels is related to same-day hospitalizations due to cardiovascular diseases across all adult age groups. The increased risk of hospitalization is small, but statistically significant, and does not persist in the week following exposure. Further data are required to confirm whether similar findings exist in people residing more locally to cities of plantation burning. These findings should inform public health policy in regions affected by sugarcane industry.

These issues cannot be assumed to be the same as those of major urban centers such as São Paulo city, since the cause of air pollution is different. In such cities, the heavy traffic influx predisposes to prolonged exposure of fossil fuels related to traffic movement in and out of town.

This vehicular-related air pollution exposure poses direct risks to the health of local residents, and, in São Paulo, is already known to be associated with hospitalizations due to cardiovascular disease in adult males^17^.

The adverse effects of sugarcane burning in Brazil have been previously reported. In a study from the city of Araraquara, Arbex et al.^1^ found a positive relationship (r = 0.238, p < 0.001) between daily variation in relatively low concentrations of Total Suspended Particulate (TSP) generated after the period of sugarcane burning and daily hospitalizations due to hypertension. This relationship was significant even when no sugarcane burning took place. In contrast to the findings of the present study, however, the study observed the effect to be sustained from the first day after TSP rise to the third day after exposure (i.e., a maintained effect)^1^.

Data from other regions of São Paulo state further support the main study findings. A study from São Paulo city^16^ showed a positive relationship between exposure to excessive air pollution (CO, PM_10_, ozone [O_3_], NO_2_, SO_2_) and same-day hospitalizations related to cardiovascular disease among older adults. For hospitalizations due to congestive heart failure, the mean risk increase was 3.17% (95%CI 2.09–4.25), while for cardiovascular-related diseases this was 0.89% (95%CI 0.18–1.61). These effects were predominantly acute (i.e., hospitalization on the same day of air pollution exposure) and occurred mostly in females^17^. Increased susceptibility of females to air pollution (excessive NO_2_) has also been reported in the United Kingdom^15^, suggesting that some negative health risks may be greater in specific population sub-groups than the general population – an important consideration for public health policy makers. Findings from Southwest São Paulo corroborate findings from the Brazilian city Santo André, where a significant rise in hospitalizations of older adults due to congestive heart failure has also been observed following PM_10_ exposure (mean increase 3.8% [95%CI 0.4–7.2])^16^.

The precise rationale to explain the findings of our study was not explicitly investigated. However, data from studies on this Brazilian region show a similarity with our study. Chiarelli et al.^6^ found a small, but significant positive association between air pollutant exposure (PM_10_ and O_3_) and increased arterial blood pressure (mean increase 2.53 mmHg [95%CI 1.25–3.80])^7^. Importantly, the effect due to O_3_ exposure was rapid, occurring between two and four hours after exposure. Ozone levels continued to rise to an increase of 49.0 µg/m^3^ by five hours, while the effect of PM_10_ remained unchanged during three hours (lag 3h). This suggests that the observed increases in cardiovascular-related hospitalizations may be caused by or at least mediated by direct, acute haemodynamic responses following environmental exposure. Other factors, however, may also interact with this process, and clarification of the precise mechanism underpinning this relationship is important.

Interesting data that further our understanding of this relationship recently emerged from Cubatão, a city 40 km from São Paulo city. Nardocci et al.^17^ investigated the impact of air pollution (PM_10_, O_3_, NO_2_, SO_2_) and meteorological variables upon hospitalizations due to cardiovascular and respiratory diseases. A robust relationship between exposure and hospitalizations was observed not only for adults (> 39 years) with cardiovascular disease, but also those with respiratory disease, suggesting that effects are strongly related to underlying respiratory pathology. This negative health outcome, however, occurred despite yearly average PM_10_ levels in this urban area being lower than those of São Paulo city at the time of the study and within the recommended air quality test of 50 µg/m^3^ established by *Conselho Nacional do Meio Ambiente* (CONAMA – National Environment Council)^6^. Similar observations of negative health outcomes associated with acceptable air pollution levels have been observed elsewhere, with individuals who have more prolonged exposure times identified as a subgroup susceptible to heightened risk^13^. This potentially important factor was unexplored in the present study; however, no relationship suggestive of susceptibility to more prolonged air pollution exposure time was observed in our data. Therefore, it may be reasonable to consider our findings a conservative underestimation of the true health impact of air pollutant exposure in Western São Paulo. Further studies appear indicated to elucidate these precise mechanisms to aid our understanding of this important issue.

The present study findings highlight the negative impact of air pollution on acute cardiovascular health in the developing region of Western São Paulo. The predominance of sugarcane industry with its associated plantation burning and increased NO_2_ levels makes this an important region for further investigation to unravel the precise mechanisms underpinning this relationship. Such information is crucial to optimise the health of residents living both locally and afar. In addition to existing evidences, these findings reinforce the necessity for banning sugar cane burning.

## Footnotes

a Instituto Brasileiro de Geografia e Estatística. 2010 Population Census. Rio de Janeiro: IBGE; 2011 [cited 2016 Nov 11]. Available from: http://www.ibge.gov.br/english/estatistica/populacao/censo2010/
